# A COMMUNITY APPROACH TO REDUCE AND PREVENT ELDER ABUSE

**DOI:** 10.1093/geroni/igae098.0145

**Published:** 2024-12-31

**Authors:** Sarah Marrs, Shannon Arnette, Madeline McIntyre, Aisling Clardy

**Affiliations:** Virginia Commonwealth University, Richmond, Virginia, United States; Virginia Commonwealth University, Richmond, Virginia, United States; Virginia Commonwealth University, Richmond, Virginia, United States; Virginia Commonwealth University, Richmond, Virginia, United States

## Abstract

Research on elder mistreatment mostly focuses on improving individuals’ skills in recognizing, screening, and reporting abuse that has already occurred, typically considered secondary prevention. This is critical for effective and timely intervention. However, increasing awareness of and accessibility to resources and supports that safeguard older adults is an effective and necessary strategy for primary prevention. Unlike child abuse victims and their families, who have access to various wrap-around resources and services, these resources are highly limited for older adult victims. The purpose of this study was to conduct focus groups with older adults, caregivers of older adults, and various aging service providers to inform the development of the Safety Connector, an online tool designed to link older adults, caregivers, and professionals to services, resources, and educational materials to prevent elder mistreatment. A mock-up of the Safety Connector was created by the research team and presented during focus groups to garner feedback and perceptions of usability and effectiveness. Findings revealed a need for simplicity, accessibility and clarity on the purpose and intended users of the Safety Connector; and to distinguish it from similar websites. The resulting Safety Connector has been integrated into our state’s existing No Wrong Door suite of websites. In this session, we will discuss research methods and results, and introduce the Safety Connector. We will also showcase other resources available on the Safety Connector’s website and discuss this as a model for linking systems of care beyond our home state.

